# Development of a highly efficient virus-free regeneration system of *Salvia miltiorrhiza* from Sichuan using apical meristem as explants

**DOI:** 10.1186/s13007-022-00872-4

**Published:** 2022-04-18

**Authors:** Si Cheng Yao, Yuan Yuan Jiang, Su Ni, Long Wang, Jun Feng, Rui Wu Yang, Li Xia Yang, Qiu Yan Len, Li Zhang

**Affiliations:** 1 College of Science, Sichuan Agricultural University, Ya’an, 625000 China; 2 Featured Medicinal Plants Sharing and Service Plantform of Sichuan Province, Ya’an, 625000 China; 3 College of Agriculture, Sichuan Agricultural University, Chengdu, 610000 China

**Keywords:** *Salvia miltiorrhiza*, Virus-free, Regeneration, Callus-organogenesis, Hydroponic

## Abstract

**Bcakground:**

The dry root and rhizome of *Salvia miltiorrhiza* are used to treat cardiovascular diseases, chronic pain, and thoracic obstruction over 2000 years in Asian countries. For high quality, Sichuan Zhongjiang is regarded as the genuine producing area of *S. miltiorrhiza.* Given its abnormal pollen development, *S. miltiorrhiza* from Sichuan (*S.m.*-SC) relies on root reproduction and zymad accumulation; part of diseased plants present typical viral disease symptoms and seed quality degeneration. This study aim to detected unknown viruses from mosaic-diseased plants and establish a highly efficient virus-free regeneration system to recover germplasm properties.

**Results:**

Tobacco mosaic virus (TMV) and cucumber mosaic virus (CMV) were detected from mosaic-diseased plants. Primary apical meristem with two phyllo podium in 0.15–0.5 mm peeled from diseased plants were achieved 73.33% virus-free rate. The results showed that the medium containing MS, 0.5 mg/L 6-BA, 0.1 mg/L NAA, 0.1 mg/L GA_3_, 30 g/L sucrose and 7.5 g/L agar can achieve embryonic-tissue (apical meristem, petiole and leaf callus) high efficient organogenesis. For callus induction, the optimal condition was detected on the medium containing MS, 2 mg/L TDZ, 0.1 mg/L NAA by using secondary petiole of virus-free plants under 24 h dark/d condition for 21 d. The optimal system for root induction was the nutrient solution with 1/2 MS supplemented with 1 mg/L NAA. After transplant, the detection of agronomic metric and salvianolic acid B content confirmed the great germplasm properties of S.m.-SC virus-free plants.

**Conclusions:**

A highly efficient virus-free regeneration system of *S.m.-*SC was established based on the detected viruses to recover superior seed quality. The proposed system laid support to control disease spread, recover good germplasm properties in *S.m.-*SC.

**Supplementary Information:**

The online version contains supplementary material available at 10.1186/s13007-022-00872-4.

## Introduction

*Salvia miltiorrhiza* Bunge, also called Danshen, is a perennial herb belonging to the family Lamiaceae and has been used to treat menstrual disorders, cardiovascular diseases, chronic pain, and thoracic obstruction over 2000 years in Asia countries [1–4]. At present, the annual consumption of medicinal materials exceeds 80,000 tons.

Due to the huge market demand, the natural population of *S. miltiorrhiza* has been drastically harvested and severely damaged. *S. miltiorrhiza* has been gradually domesticated and induced to various places in China, such as Sichuan, Shandong, Hebei, etc. [1, 5]. In particular, *S. miltiorrhiza* produced from Zhongjiang of Sichuan (*S.m.*-SC) is considered the best-quality product for stick, bright red, and high content of salvianolic acids in the roots; it has been exported overseas, and Zhongjiang is deemed as genuine producing area [5, 6]. However, the pollen of *S.m.*-SC develops abnormally and relies on root reproduction [7]. With disease accumulation in cultivated populations of *S.m.*-SC, the quality and quantity of diseased plants have declined seriously [8]. Diseased plants of *S.m.*-SC present whitening, mottled and yellow leaves, and premature withering, and some plants present typical symptoms of viral disease. Ding et al. reported that *cucumber mosaic virus* (CMV) infected *S.m.*-HB (*S. miltiorrhiza* produced from Hebei province, China) with symptoms of mosaic, stunting, chlorosis, and mottle [9]. No research has reported on virus in *S.m.*-SC. The unknown virus of diseased *S.m.*-SC must be detected for disease prevention and control.

Virus elimination in vitro plant culture is the most effective approach to obtain virus-free germplasm resources of superior variety to be applied to many crops, such as *Solanum tuberosum* [10, 11]. Virus-free regenerated plants grow fast and have strong disease resistance ability and significantly improved production and quality [12]. Few regeneration systems have been established through callus genesis for *S. miltiorrhiza* [13–15]. All of these systems use the seeds or vegetable organ (leaf, root, etc.) as the starting materials but cannot remove zymad from the plant, explants, and callus, showing browning or vitrification; moreover, the salvianolic acid B content in the regeneration plant is low. None of the systems are suitable for in vitro preservation, purification, and rejuvenation of *S.m.*-SC plants. A highly efficient virus-free regeneration system for *S.m.*-SC remains to be developed.

Overall, the current study was aim to detect the virus of diseased plant and development a high efficient virus-free regeneration system for *S.m.*-SC to recover good germplasm properties and improve its yield and quality.

## Results

### Virus detection and symptom observation of diseased plants

**Fig. 1:**
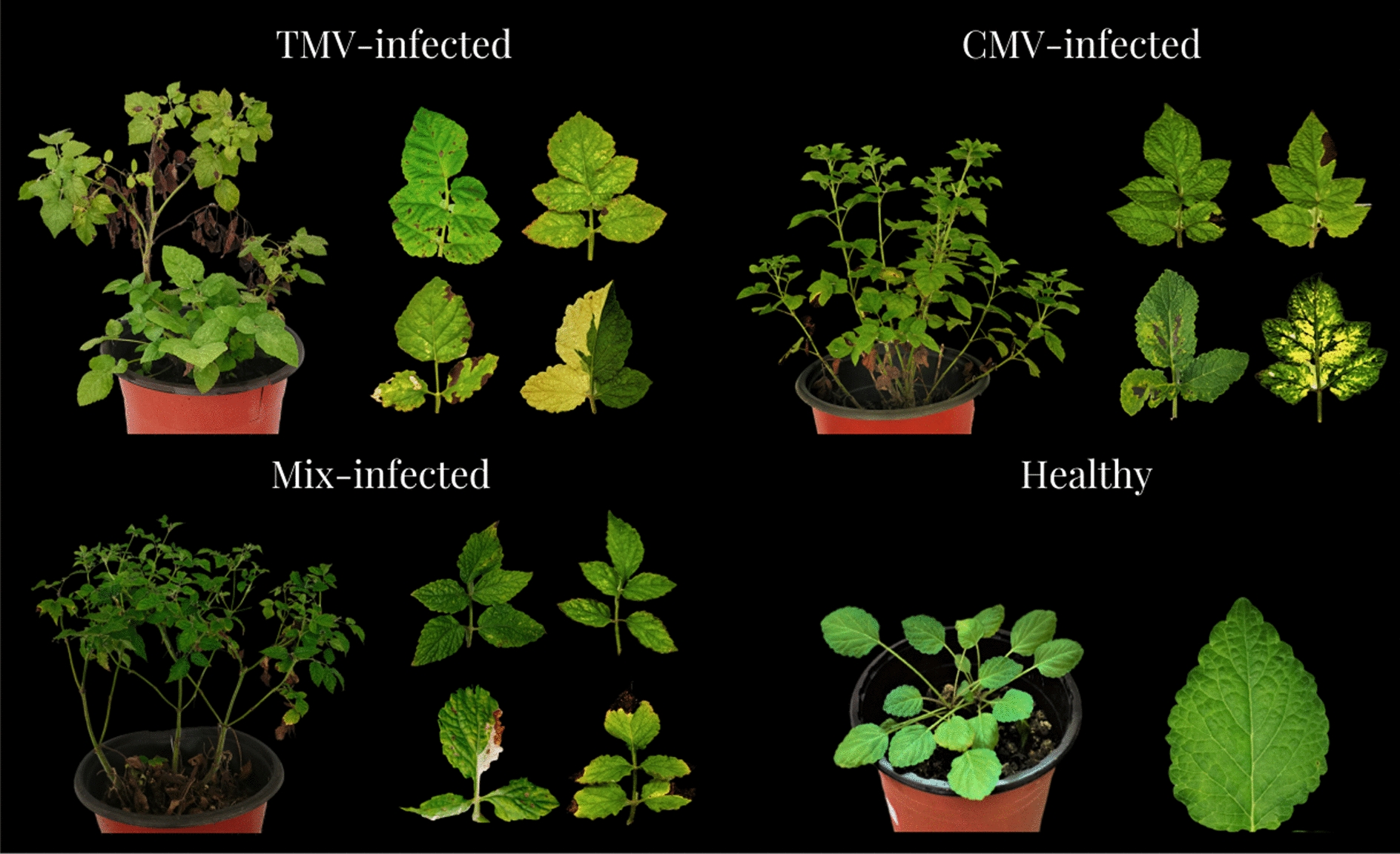
Morphological expression of virus-infected plants and healthy plant of *S.m.*-SC. TMV-infected: *Tobacco mosaic virus* (TMV) positive by Das-ELISA; CMV-infection: *Cucumber mosaic virus* (CMV) positive by Das-ELISA; Mix-infected: positive of TMV and CMV by Das-ELISA at the same time

From field trait observation, 17 diseased plants were selected to serologically test for *Cucumber mosaic virus* (CMV), *Tobacco mosaic virus* (TMV), *Tomato mosaic virus* (To MV), and *Tomato spot wilt virus* (TSWV) Das-ELISA (Double antibody sanwich-enzyme linked immunosorbent assay). Of the 17 plants, 9 were positive for TMV, 2 were positive for CMV, and 3 had mixed infection with TMV and CMV. None of the samples was found positive for To MV or TSWV, and the total virus-infection incidence was 82.35% (Table 1, Fig. 1).

**Table 1:** Virus detection and symptom observation of diseased plants of *S.m.*-SC by Das-ELISA test

Plant	Virus				Symptom of diseased plant
	TMV	CMV	To MV	TSWV	
D1	P*	N	N	N	(1) Mosaic; (2) Albino in young leaf; (3) Vein clearing; (4) Branches wither prematurely
D2	P*	N	N	N	
D3	P*	N	N	N	
D4	P*	N	N	N	
D5	P*	N	N	N	
D6	P*	N	N	N	
D7	P*	N	N	N	
D8	P*	N	N	N	
D9	P*	N	N	N	
D10	N	P*	N	N	(1) Yellow mosaic spreading from vein; (2) Bulk dry spot
D11	N	P*	N	N	
D12	P*	P*	N	N	Mosaic; Black spots; Branches wither prematurely
D13	P*	P*	N	N	
D14	P*	P*	N	N	
D15	N	N	N	N	(1) Withered leave; (2) Necrotic spot, mottled
D16	N	N	N	N	
D17	N	N	N	N	

P*: positive, N: negative; all plant samples were determined at least twice

The virus-infected plants presented mosaic in mature leaf, while the leaf morphology was found to be diverse between virus-infected plants and healthy plants (Table 1, Fig. 1). The leaves of healthy plants are green and stretched, have no black spots or mosaic, and grow strongly. The TMV-infected plants show black spots, and some of the plants have half of albino leaf in young leaf. Black spots gradually develop and expand on the leaf surface as the plants grow, and the plants die eventually. The CMV-infected plants have obvious yellow mosaic symptoms that spread from the leaf vein. Black spots were also detected in some mature leaves. However, no severe symptoms in *S. m.-SC* were observed in mixed infections of TMV and CMV, which present white spot mosaic symptoms, black spot, and premature withering of mature leaves in the whole plant.

### Screening of culture medium for apical meristem growth

**Fig. 2:**
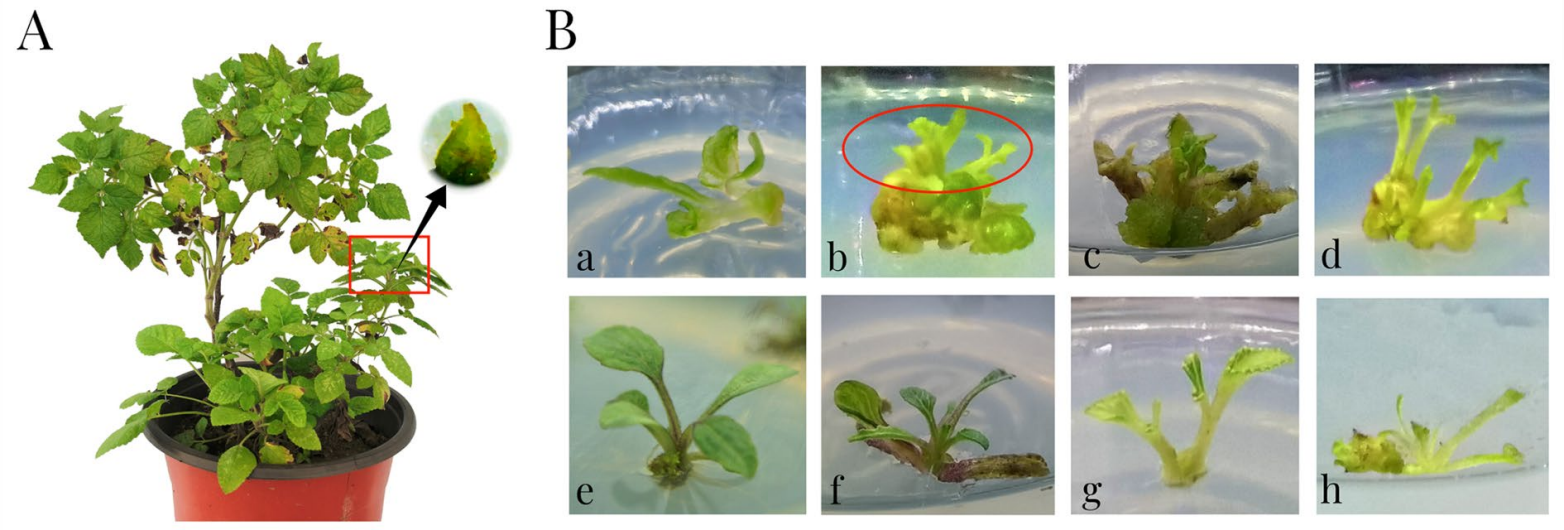
**A** Schematic drawing of primary apical meristem of *S.m.*-SC. The red box shows the terminal buds of *S.m.*-SC. The black arrow indicates the peeled primary apical meristem. **B** Growth of primary apical meristem in buds induction medium (BIM) under the light condition with 16 h light/ 8 h dark. **a** vitrified bud growing in B2 media, **b** buds growing in B3 media (the circle indicates emaciated buds), **c** vitrified bud and callus growing in B4 media, **d** emaciated, vitrified buds growing in B5 media. **e** vigorous bud growing in B6 media. **f** emaciated, vitrified bud growing in B7 media. **g** emaciated bud growing in B8 media. **h** vitrified bud growing in B9 media

The peeled primary apical meristem (PM) with two phyllo podium in 0.15–0.5 mm from virus-infected plants was used for initiated culture and detoxification treatment (Fig. 2A). The concentrations of cytokinin, sucrose, and agar were adjusted in B0–B9 groups to identify the suitable media for apical meristem growth.

Table 2 and Fig. 2B indicate that the media components significantly influenced the survival of primary meristem. Exogenous cytokinin (6-BA) has a crucial effect on the growth of apical meristem. When 6-BA was used in the media, the survival rate significantly increased compared with that in the control, resulting in the lowest survival rate of 31.82% in B0 media to the highest survival rate of 75.47% in B3 media (supplied with 0.5 mg/L 6-BA), while the bud was emaciated and vitrified. The concentrations of sugar and agar were adjusted in B5–B9 medium at the same exogenous cytokinin level. The result shows that the survival rate of primary meristem significant decreased with increasing agar concentration in B5, B7, and B8 medium. The buds were emaciated or vitrified (Fig. 2B: d, f, g). At mean time, increasing the concentration of sucrose was beneficial to the survival of primary meristem by comparing the 30 g/L group (B3, B6 and B9 medium) and the 20 g/L group (B5, B7, and B8 medium).

**Table 2:** Effect of medium components in combination with MS medium on the survival of apical meristem

Group	Medium components					Survival Rate (%)	Growing status
	NAA (mg/L)	GA_3_ (mg/L)	BA (mg/L)	Agar (g/L)	Sucrose (g/L)		
B0	0	0	0	8.5	30	34.09 ± 4.69 ^d^	–
B1	0.1	0.1	0	8.5	30	40.48 ± 4.41 ^d^	–
B2	0.1	0.1	0.25	8.5	30	67.35 ± 4.24 ^b^	Vitrified bud
B3	0.1	0.1	0.50	8.5	30	75.47 ± 2.53 ^a^	Partial health buds
B4	0.1	0.1	1.00	8.5	30	58.00 ± 2.81 ^c^	Vitrified buds, callus formed
B5	0.1	0.1	0.50	7.5	20	75.47 ± 2.89 ^a^	Emaciated, vitrified buds
B6	0.1	0.1	0.50	7.5	30	77.36 ± 2.61 ^a^	Vigorous bud
B7	0.1	0.1	0.50	8.5	20	62.50 ± 3.37 ^b^	Emaciated bud
B8	0.1	0.1	0.50	9.5	20	53.06 ± 5.65 ^c^	Emaciated bud
B9	0.1	0.1	0.50	9.5	30	69.44 ± 4.34 ^b^	Vitrified bud

Number of apical meristem inoculated per group was twelve; three replication of each group were laid. Data were collected after 4 weeks. Different letters indicate significant differences among mediums for each group as determined by Duncan’s multiple range test followed by one-way ANOVA at the p < 0.05 significant level

The highest survival rate was 77.36% in B6 media (Table 2), and vigorous directly regenerated shoot was obtained after culture (Fig. 2B: e). The virus carrying states of 45 directly regenerated shoots from 15 primary apical meristems trimmed from five virus-infected plants were tested, and the virus-free rate was 73.33% (Figs.2).

Therefore, the B6 media containing MS + 0.1 mg/L NAA + 0.1 mg/L GA_3_ + 0.5 mg/L 6-BA + 30 g/L sucrose + 7.5 g/L agar was selected for the initial culture of primary apical meristem.

### Callus induction from directly regenerated tissue

**Fig. 3:**
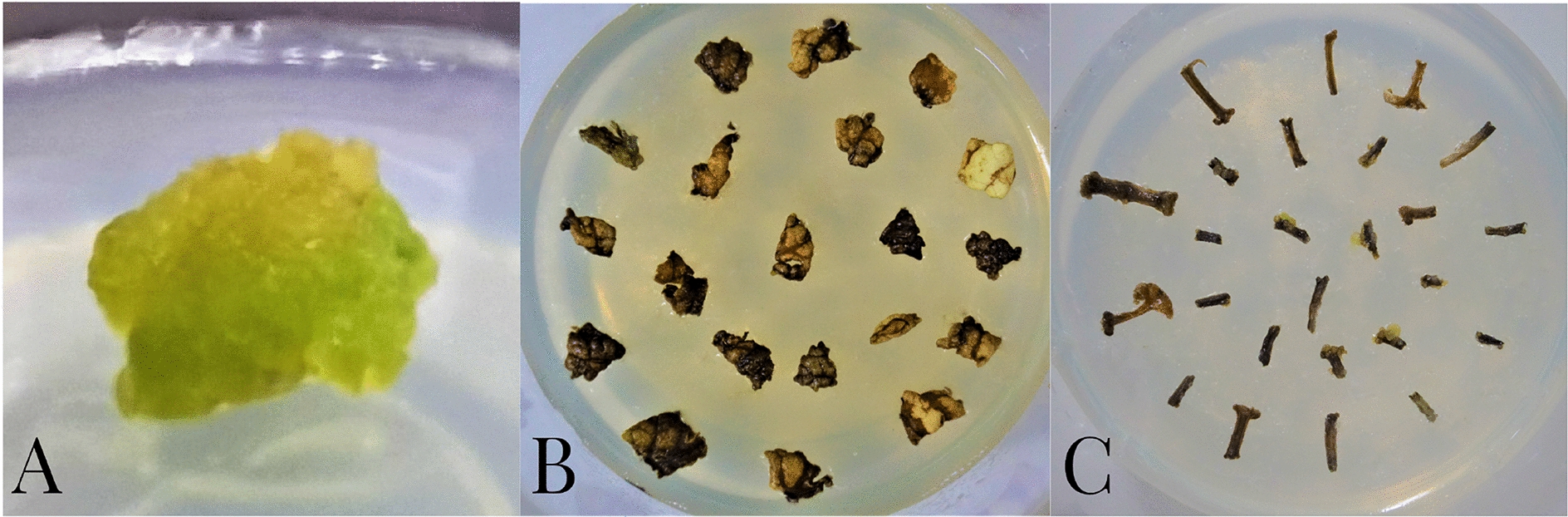
Callus growth of different secondary materials (cutted from directly regenerated buds) on B6 media under the light condition with 16 h light/ 8 h dark. **A** Callus from secondary apical meristem, **B** culture effect of secondary leaf in B6, and **C** culture effect of secondary petiole in B6. Three replication of each group were laid. Data were collected after 3 weeks

Secondary apical meristem, leaf and petiole were took up from virus-free directly regenerated buds obtained by detoxification culture to induce callus. The results show that the yellow-greenish callus was induced from the secondary apical meristem (SM) without phyllo podium (Fig. 3A). Most of secondary leaf (SL) and secondary petiole (SP) manifest browning and have poorly induced callus under the PM growth condition (Fig. 3B, C).

The inducing effect of the six media for three cytokines on callus formation under two light conditions (16 h light/8 h dark and 24 h dark/day) was investigated using secondary leaf and petiole to optimize the induction condition for callus formation. L0 (without exogenous cytokines) was used as control.

**Fig. 4:**
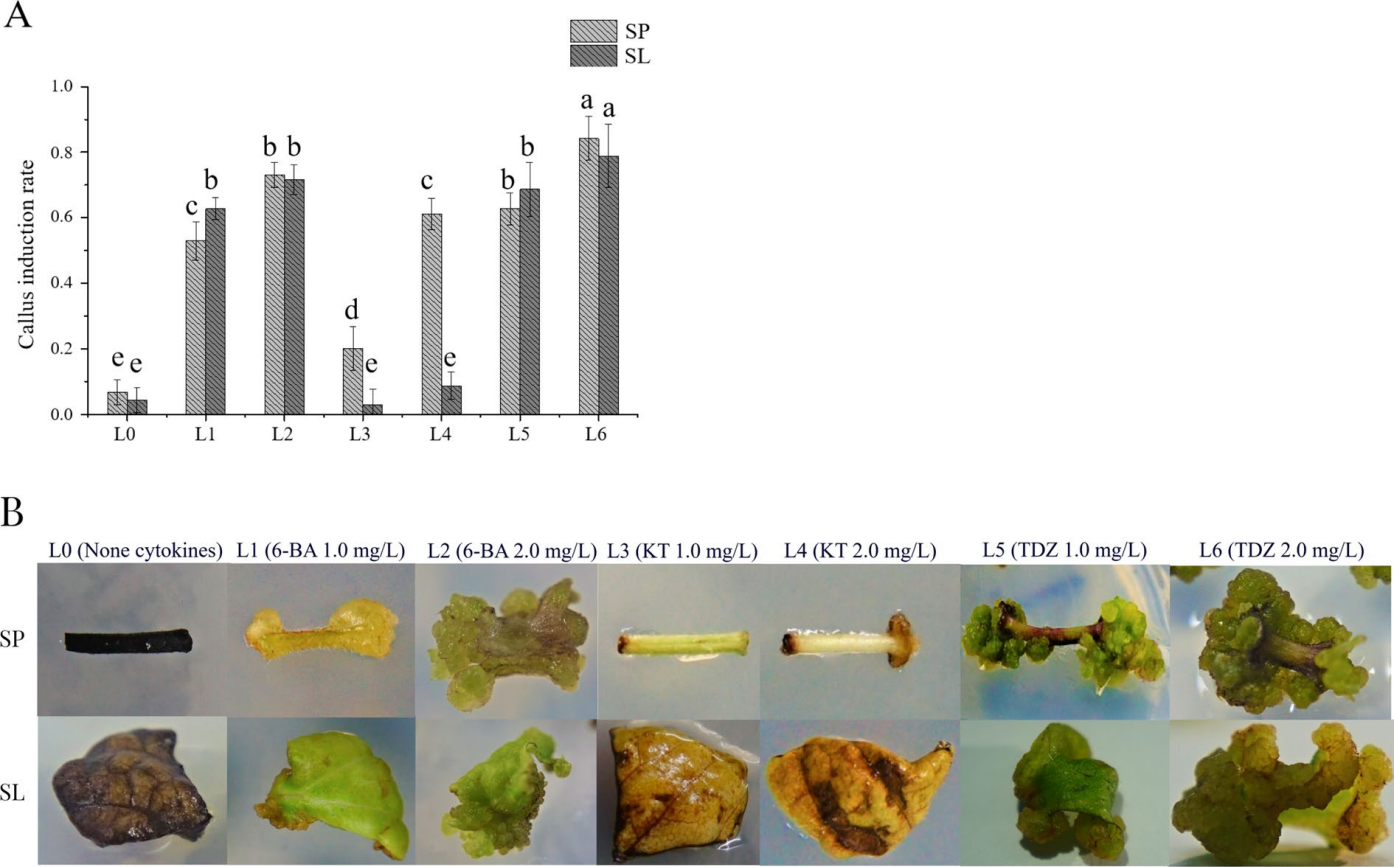
Effect of different kinds and concentrations of cytokines on callus induction under 24 h dark/days light condition. **A** Induction rate of callus from SP and SL on 24 h dark/d light condition in L0-L6 media. **B** Induction effect of SL and SP in L0-L6 media under 24 h dark/d. SP: secondary petiole; SL: secondary leaf. Different letters indicate significant differences among mediums for each group as determined by Duncan’s multiple range test followed by one-way ANOVA at the P < 0.05 significant level. Three replication of each group were laid. Data were collected after 3 weeks

Significant difference (P < 0.05) in the main effect of light and exogenous hormone kinds was observed in the formation of secondary callus (Figs. 3 and 4). Overall, secondary leaf has higher callus-genesis potential, with the induction rate of 73.32%. Light significantly represses the formation of secondary callus. When the light condition changed, the callus induction rate (33.33%–45.90%) of secondary leaf and petiole under 16 h light/8 h dark condition significantly increased to 57.4%–73.32% under 24 h dark/d. The induction varied rate of leaf callus was 40% compared 16 h light/8 h dark with 24 h dark, and that of petiole was 12% only, indicating that leaf was more sensitive to light condition than petiole (Fig. 3A). Under the light condition of 16 h light/8 h dark, most explants appeared as drying and browning after 30 days, and poorly grown brown callus was also induced. The browning callus from petiole and leaf developed hyperhydric shoots, or growth was stopped after 40 days of induction. The leaf and petiole product, namely, yellow–greenish callus was obtained from the incision under 24 h dark/day, and the growth was normal, and browning was not observed (Fig. 3B).

The influence of different kinds and concentrations of cytokines for callus induction from petiole and leaf explants was measured under the light condition of 24 h dark/d (Fig. 4A, B). With increasing hormone concentration, the callus induction rate significantly increased. On media L3 and L4 (supplied with 1 or 2 mg/L KT), explants were yellowish-brown, scarce callus formation (2.78%–8.70%) was observed in the leaf or browning callus was formed at the petiole incision. By contrast, on the media supplemented with 6-BA or TDZ, 52.94%–84.21% of the explants produced callus. The highest induction rate (84.21%) was obtained on the media supplemented with 2 mg/L TDZ for secondary petiole callus induction, and the callus was compact and greenish-yellow.

The optimized light and medium condition for secondary callus induction is that cutting secondary petiole into 10 mm length and inducing in the media containing MS + 2 mg/L TDZ + 0.1 mg/L NAA + 30 g/L sucrose + 7.5 g/L agar. The culture was kept for 3 weeks under 24 h dark/d light condition.

### Induction and growth of adventitious buds

Callus reached the differentiation phase of organ after almost one month of vegetative growth phase. Callus from secondary apical meristem, secondary leaf, and secondary petiole fragment was inoculated in screened media (Table 2, B6) to induce bud organogenesis.

**Fig. 5:**
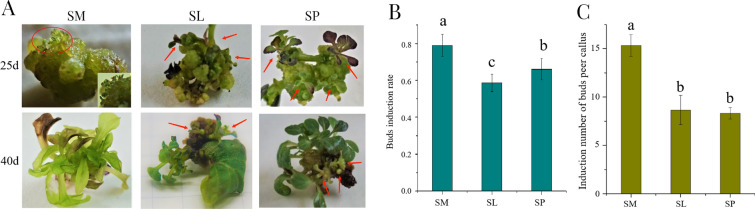
**A** Bud organogenesis of *S.m.*-SC through secondary callus on B6 media under the light condition with 16 h light/8 h dark. **B** Regenerated buds' induction rate of callus. **C** Induction number of regeneration buds per callus. SM: Buds induced from secondary apical meristem, the growth status of buds: long and narrow leaf, transparent yellow green, vitrification (Small image on the lower right shows buds organogenesis in red circle). SL: buds induced from secondary leaf, the growth status of buds: small buds slowly product from the spherical embryoid growing from the surface bulge of massy callus (arrow indicates bud organogenesis). SP: buds induced from secondary petiole, the growth status of buds: vigorous, green leaf stretch (arrow indicates bud organogenesis). Different letters indicate significant differences among mediums for each group as determined by Duncan’s multiple range test followed by one-way ANOVA at the p < 0.05 significant level, and all of materials was repeated three times

The formation of adventitious buds was an event observed in the callus of different origins, did not all occur healthy buds after 40 days of culture (Fig. 5). The regeneration ability of secondary apical meristem was examined. Hyperhydric buds were produced from the pale yellow-green callus (Fig. 5A: SM). The callus from apical meristem had better plant regeneration ability, as verified in terms of regeneration rate and growth coefficient. The callus of secondary apical meristem with an average of 15.33 buds/explant and 78.86% regeneration rate, has a greater regeneration potential for buds organogenesis, compared with secondary leaves and petiole (Fig. 5B, C). This finding indicated that tender tissue was sensitive to medium component and negative to healthy bud proliferation. Regarding the development of secondary leaf callus, adventitious buds were produced from spherical embryoid growing from the surface bulge of massy callus (Fig. 5A: SL). However, vigorous adventitious buds were produced on the callus of petiole incision. The structure had a deeply yellow-green dense callus, and the inner is lignified (Fig. 5A: SP). The numbers of adventitious buds per callus of secondary leaf and secondary petiole were 8.33 and 8.67, and the regeneration rates were 58.61% and 66.03%, respectively (Fig. 5B, C).

In short, apical meristem is efficient in micro-propagation because of its high regeneration frequency. Embryonic callus has compact structure in deep yellow-greenish color in in vitro culture for *S.m*-SC. Thus, the convenience and regeneration ability of explant were comprehensively considered. Secondary petiole callus was selected as the material for high-efficient adventitious bud induction.

### Root induction and plant acclimatization

**Fig. 6:**
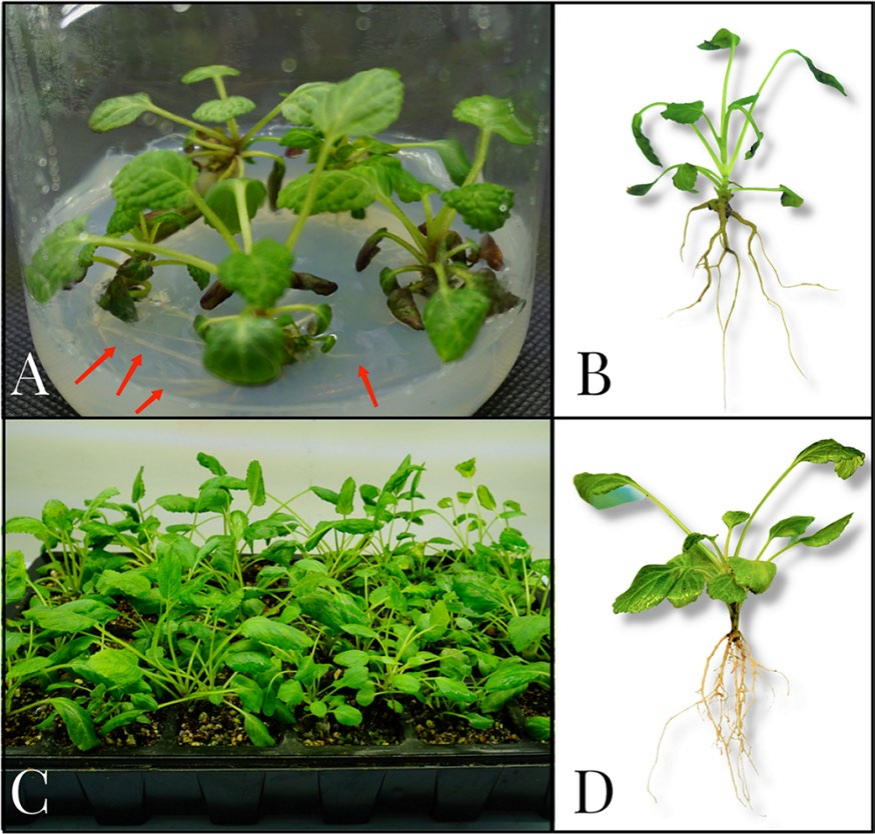
Growth of virus-free regenerated plants of *S.m.*-SC under the solid medium and improved hydroponics. **A** Growth of virus-free regenerated plants in M3 group. Arrow indicates the regenerated roots. **B** Growth effect after transplantation of the virus-free regenerated plant rooted in M3 group. **C** Growth of virus-free regenerated plants in H4 group. **D** Growth effect after transplantation of the virus-free regenerated plant rooted in H4 group

Root regeneration is a crucial step for successful regeneration of plants. The quality of the adventitious roots directly affects subsequent acclimatizati*o*n and transplant survival rate. When the regeneration buds were dissected into single bud and grew to 3–5 cm high in the media, adventitious roots were induced in the solid medium and improved hydroponics. Two styles for nine combinations were designed for root regeneration of *S.m.*-SC. The plant growth effect is presented in Table 3 and Fig. 6.

**Table 3:** Effect of the different induction styles and nutrient components on the root regeneration of *S.m.*-SC

Induction style	Group	Basel medium	IBA (mg/L)	NAA (mg/L)	Sucrose (g/L)	Rooting rate (%)	Main Root number	Main Root Length(cm)	Main Root diameter(mm)
Solid medium	M0	1/2 MS	–	–	10	38.46 ± 4.36 ^f^	2.33 ± 0.58 ^b^	3.47 ± 0.93 ^f^	0.70 ± 0.06 ^b^
	M1	1/2 MS	0.5	0.5	10	53.33 ± 6.67 ^e^	1.67 ± 0.58 ^c^	3.41 ± 1.01 ^f^	0.67 ± 0.09 ^b^
	M2	1/2 MS	1.0	–	10	74.42 ± 3.70 ^c^	5.33 ± 1.15 ^a^	4.48 ± 1.46 ^e^	0.87 ± 0.03 ^b^
	M3	1/2 MS	–	1.0	10	80.00 ± 6.67 ^b^	4.00 ± 0.84 ^a^	4.14 ± 0.75 ^f^	0.91 ± 0.05 ^a^
Improved hydroponics	H0	–	–	–	0	60.98 ± 2.10 ^d^	2.33 ± 0.58 ^b^	6.71 ± 0.19 ^e^	0.40 ± 0.06 ^d^
	H1	1/2 MS	–	–	0	75.50 ± 4.41 ^c^	2.67 ± 0.58 ^b^	9.89 ± 0.35 ^b^	0.53 ± 0.05 ^c^
	H2	1/2 MS	0.5	0.5	0	83.33 ± 3.30 ^a^	3.67 ± 0.58 ^b^	7.64 ± 1.01 ^d^	0.33 ± 0.03 ^d^
	H3	1/2 MS	1.0	–	0	72.22 ± 1.92 ^c^	5.00 ± 0.10 ^a^	10.86 ± 0.77 ^a^	0.44 ± 0.07 ^c^
	H4	1/2 MS	–	1.0	0	87.88 ± 6.67 ^a^	4.00 ± 1.34 ^a^	8.47 ± 1.17 ^c^	0.55 ± 0.09 ^c^

^a^ For each group (M0-H3) and rooting affection rooting rate, main root number, length, and diameter means separated by difference letter are significantly different at P < 0.05 according to Duncan's test. Number of regeneration buds per group was twelve; three replication of each group were laid

The rooting effect was compared among M0–M3 groups. The result showed that the regenerated plants grew slowly, malnutrition occurred, and the lowest rooting rate (38.46%) was found in the control group. Under the same auxin concentration (1 mg/L), applying a single auxin in the medium had better root induction effect than mixed use. The M3 group supplied with 1 mg/L NAA had greater rooting rate (80.00%) than M1 and M2. In the H0 group without added nutrients and auxin, the plants grew slowly and the rooting rate was lower. After adding the basal medium, the plants grew vigorously, and the rooting rate was significantly increased to more than 70%. The H4 group had the best growth effect, and the rooting rate reached 87.88%. Based on comparison of the solid medium and improved hydroponics, the roots induced by hydroponics were thinner and longer (Table 3).

After hard-seeding in nutrient soil for 21 days, the virus-free regenerated plants were transplanted into soil. The growth effect and survival rate after the transplant of the regenerated plants were compared between M3 and H4 groups. The leaves of the M3 plants were easy to be dehydrated and wilt after transplanting (Fig. 6A, B), and the survival rate was only 65.29% after 30 days. However, the leaves in the H4 group plants had healthy growth, the leaves were stretched and emerald green, the root system was slender (Fig. 6C, D), and the survival rate reached 88.64% after transplanting. The leaves of whole regenerated plants were collected after rooting, and no positive reaction for TMV or CMV was detected in the Das-ELISA test.

The result showed that inducing root formation in the improved hydroponics is a suitable option to replace root induction in sterile solid media. Nutrient solution with 1/2 MS supplemented with 1 mg/L NAA and free-sugar in hydroponics can facilitate efficient plant regeneration and survival of *S.m.*-SC.

### Evaluation of yield metric and active ingredient

The whole virus-free plants were transferred to soil after acclimatization. Common plants were planted under the same condition as control. In total, 10 virus-free and common plants were collected after 10 months of culture for yield metric and main active ingredient analysis of roots.

The yield metric and active ingredient of the root of the virus-free plant significantly improved compared with those of the common plants (P < 0.05, Table 4). The average root fresh weight of virus-free plants was 398.50 ± 87.88 g/plant, and the yield was increased by 62.96%. The average main root length (250.00 ± 36.51 mm) and diameter (14.06 ± 1.84 mm) of the virus-free plants 9were improved by 25% compared with those in the common plants. The main root number was not significantly different (P > 0.05) between virus-free plants and common plants.

**Table 4:** Yield metric and salvianolic acid B content (mean ± SE) of root for virus-free plants and common plants of *S.m.*-SC after 10 months of culture (n = 10 for virus-free plants and common plants, respectively)

Plants	Root Weight (g/plant)	Main root Number	Main root Length (mm)	Main root Diameter (mm)	Salvianolic acid B (mg/g)
Virus-free	398.50 ± 87.88 ^a^	6.60 ± 1.82 ^a^	250.00 ± 36.51 ^a^	14.06 ± 1.84 ^a^	87.87 ± 14.74 ^a^
Common	244.54 ± 133.95 ^b^	6.46 ± 1.96 ^a^	200.81 ± 58.85 ^b^	11.20 ± 2.73 ^b^	67.29 ± 16.66 ^b^

^a^ Means with different letters are significantly different at P < 0.05 according to Student's t-test. Three replication of each plants were laid. Yield data were obtained by measuring fresh roots after harvest, and salvianolic acid B content was measured after drying

Salvianolic acid B is one of the main biologically active secondary metabolites in the dry roots and rhizomes of *S. miltiorrhiza* [16]. The HPLC analysis showed that the dry root of the virus-free plants had 87.87 ± 14.74 mg/g salvianolic acid B, and that of the common plants had 67.29 ± 16.66 mg/g. The values represented a significant increase (P < 0.05) by 30.58% in the dried roots in the virus-free plants compared with those in the common plants.

Virus-free plants grow robustly, the main roots are thick and long, the yield is high, and salvianolic acid B content is significantly increased (P < 0.05).

## Discussion

### Virus-free and regeneration of apical meristem

The selection of suitable explant is the base of success for in vitro culture. Plant meristem has remarkable regenerative and antiviral capacity, enabling them to either recover damage tissues or established de novo organ [17, 18]. Wu et al. reported that the stem cell regulator WUSCHEL was expressed in response to viral infection and inhibited its accumulation to protect the central and peripheral areas in *Arabidopsis* meristem [19]. However, apical meristem isolation is very time consuming, and injury or bruising during lengthy isolation can usually kill the meristem [20]. Cytokinin enhances the mitotic activity of the stem cell to reduce the damage by disinfection and splicing of explants. The survival rate of primary apical meristem of *S.m.*-SC was increased by improving the concentration of cytokinin. Isah et al. demonstrated that cytokinin promoted the growth of *Gymnema sylvestre* meristem, which turned green earlier, and increased the survival rate significantly [21]. During in vitro culture, endogenous and exogenous plant hormones can trigger cellular reprogramming, and osmotic pressure and hardness significantly affect explant survival and regeneration [22]. In the present study, the apical meristem on high-hardness medium presented with vitrification symptom, and the survival rate was improved by increasing the sugar concentration. Hence, in the construction of in vitro culture environment, the key factors of growth hormones should be given attention, and the adjustment of other factors, such as osmotic pressure and hardness, should not be ignored.

### Effect of PGR and light during tissue culture

The ratio of auxin and cytokinin directs the differentiation progress of explants in in vitro culture [23]. A high concentration of cytokinin promotes callus formation. The formation of embryogenic tissue is the key to regeneration. Wounding stress induces the local accumulation of cytokinin at the wound site and modifies hormone biosynthesis, thereby activating the transcription and translation processes and leading to changes in cell cycle phase and callus formation [22]. In early stage of red maple in in vitro culture, callus induction and embryo were more dependent on PGR, especially TDZ [24]. In this article, TDZ showed better embryonic callus induction ability of *S.m.*-SC compared with 6-BA and KT, consistent with the result reported by Chen [13]. Although hormone effects are stage specific, the medium containing TDZ only was not effective for bud regeneration or induced vitrification and emaciation of apple buds; high shoot regeneration was achieved in the media supplied with 6-BA only [25]. Here, vigorous buds were induced from deeply yellow–green dense callus in B5 media supplied with 0.5 mg/L 6-BA, 0.1 mg/L NAA, and 0.1 mg/L GA_3_.

Light is one of the key factors that affect plant regeneration and growth. Callus genesis and shoot formation are suppressed by light in vitro culture of apple [25, 26]. Here, browning explants and poor callus genesis were observed during 16 h light/8 h dark condition after 30 days of culture of *S.m.*-SC, which may be caused by the oxidation of abundant photosensitive substances (phenolic acids, flavonoids, and tanshinones) in the explants [27, 28]. Change in light condition is essential during redifferentiation and bud growth stage, and dark pre-treatment of the explant for 3 weeks under 16 h light/8 h dark condition enhanced the formation of apple shoots [25]. After dark culture for a month, pale shoots with elongated stem and lack of chlorophyll in the apex were detected after in vitro regeneration of eggplant [29]. The seedlings of *S.m.*-SC show slender stems, yellow–green leaves, and vitrification under long-term dark conditions.

### Rooting and acclimatization of regenerated buds

In regenerated plants under in vitro condition, the surface of leaf lacks cuticle, the stoma is highly opened, and transpiration is extremely strong. Changes in environment humidity and organ damage (such as leaf or root breakage) during transplantation can easily cause water loss and withering and even death [24, 30, 31]. Similarly, the regenerated plants in solid medium experienced severe water loss and wilting after transplantation, resulting in a low survival rate of only 65.29%. Meanwhile, the regenerated plants in hydroponics grew vigorously, and the survival rate reached 88.64%. The great root induction effect may be due to the fact that humid environment and good air circulation can make the regenerated plants to better adopt to the environment and growth faster [31]. Kyle et al. developed an improved axenic hydroponic propagation system to produce large quantities of the roots of *Taraxacum koksaghyz*; the plants grew faster in the system than in the solid medium [32].

### Change in the content of the main active ingredient of plants after virus-free

Salvianolic acids are the main active secondary metabolites in the dry roots and rhizomes of Danshen. Phenolics participate in plant stress resistance as constitutive expression products of genes, while ketones are considered secondary metabolites of inducible expression. For example, higher constitutive levels of total phenols were found in resistant synthetic hexaploid wheat compared with those in moderately resistant and susceptible genotypes [33]. During the development process, salvianolic acids exist in both the above-ground and underground parts of *S. miltiorrhiza* [34, 35], indicating that phenolic acids may be synthesized in both parts. After detoxification, the virus and other diseases in the plant are reduced, the growth is vigorous, the leaves are dark green, and the photosynthesis is enhanced, thereby promoting the synthesis of salvianolic acids and significantly increasing (P < 0.05) the yield of *S.m.-SC* compared with those in the common plants (Additional file 1).

## Conclusion

**Fig. 7:**
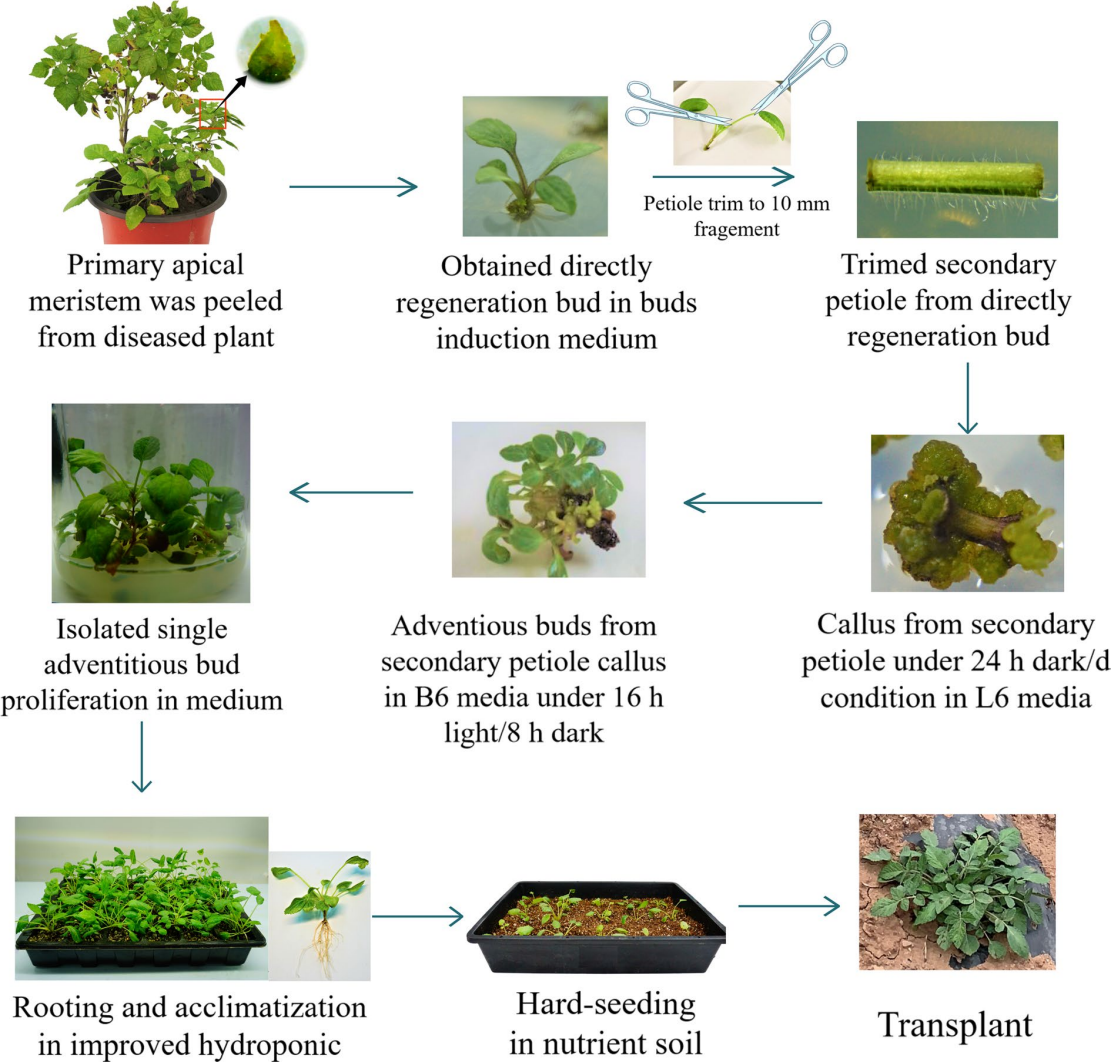
Established virus-free regeneration system for *S.m.*-SC. Starting from explant disinfection and cultivation in B6 media (MS basal medium supply 0.5 mg/L 6-BA, 0.1 mg/L NAA and 0.1 mg/L GA_3_), light condition (24 h dark/d), and medium component (MS basal medium supply 2 mg/L TDZ and 0.1 mg/L NAA) for callus induction of secondary petiole tissue. After adventitious bud induction and proliferation in B6 media, the shoots were rooting and acclimatization was improved hydroponics (culture liquwid component: 1/2 MS basal medium supply 1 mg/L NAA). The completely regenerated shoots were subjected to hardening-seeding in nutrient soil and transplanted in soil. The red box in diseased plant shows the terminal buds of *S.m.*-SC. The black arrow indicates the peeled primary apical meristem. The red circle in directly regenerated bud shows the trimming part of secondary petiole. The virus detection of regenerated plants: hen the seedlings grow to 8-10 cm height, collect directly regenerated seedlings and adventitious bud leaves for virus detection

CMV and TMV were first determined from *S. miltiorrhiza* from Sichuan diseased plants. An efficient virus-free regeneration system for *S.m.*-SC was established using apical meristem as explants for the first time (Fig. 7). In summary, the optimal bud induction media contained MS, 0.5 mg/L 6-BA, 0.1 mg/L NAA, 0.1 mg/L GA_3_, 30 g/L sucrose, and 7.5 g/L agar. For callus induction, the optimal condition was L6 media containing MS, 2 mg/L TDZ, 0.1 mg/L NAA, 30 g/L sucrose and 7.5 g/L agar and using 10 mm secondary petiole of virus-free plants under 16 h light/d condition for 21 days. For rooting induction, the optimal system was using nutrient solution with 1/2 MS supplemented with 1 mg/L NAA. After transplant, virus-free plants grew robustly, the main roots were thick and long, the root yield and salvianolic acid B content in the dry root were significantly increased to 398.50 g/plant and 87.87 mg/g by 62.96% and 30.58% compared with those in common plants.

## Materials and method

### Plant material

The diseased plants of *S. miltiorrhiza* were collected from a planting base in Zhongjiang, Sichuan, China. The whole living plants were kept in the Teaching and Research Base of Sichuan Agriculture University (Ya'an, Sichuan, China). The buds sheared from the diseased plants were selected as the experiment material.

### Virus detection of plants

Leaves collected from 17 *S. miltiorrhiza* diseased plants (marked as D1–D17, Table 1) and regenerated plants were serologically subjected to detection of viruses in Double antibody sandwich elisa (Das-ELISA). *Tobacco mosaic virus* (TMV), *Cucumber mosaic virus* (CMV), *Tomato mosaic virus* (To MV), and *Tomato spotted wilt virus* (TSWV) were detected. For ELISA tests, the reagents, buffers, and controls supplied by AGDIA (American) were used in accordance to manufacturer’s instructions. The detection of the regenerated buds is the same as that of the diseased mother plant.

### Explant sterilization and screening of bud induction medium

Parts of the plants were selected to screen the bud induction medium of *S. miltiorrhiza* by using apical meristem after virus detection of the diseased plant. Buds with no fully spread leaves sheared from the diseased mother plants were surface cleaned by detergent for 5 min and rinsed for 30 min under running water before sterilization at gnotobasis. The explants were immersed in 75% alcohol for 45 s and washed twice with sterilized distilled water. The explants were cleaned by soaking into 0.1% mercury chloride solution for 7 min and 30 s and washed six times with sterilized distilled water under aseptic condition.

Primary apical meristem (PM, Fig. 2A) was exfoliated from the sterilized plant material under digital microscope (yookdd DM3) and transferred into the bud induction media (BIM, B0–B9) containing 1 × Murashige and Skoog (MS) base medium (Chembase, China) with 0.1 mg/L 1-naphthaleneacetic (NAA) (Chembase, China) and 0.1 mg/L Gibberellin A_3_ (GA_3_) (Chembase, China) and supplied with different concentrations of 6-benzylaminapurine (6-BA, 0, 0.25, 0.50, and 1.00 mg/L) (Chembase, China), agar (7.5, 8.5 and 9.5 g/L), and sucrose (20 and 30 g/L); B0 containing 1 × MS basal salt without any phytohormone was used as control (Table 2). One apical meristem was inoculated in a glass culture bottle (65 mm × 115 mm with breathable plastic cover). About 50 mL of the medium was placed per bottle for meristem culture. Twelve meristems were inoculated in each medium, and the experiment was repeated three times. The cultivating bottles were placed in a thermostatic chamber at 22 ± 1 ℃ with a photoperiod of 16 h light/8 h dark under white fluorescent light (average illumination intensity 75 μmol m^−2^ s^−1^). The survival rate of primary apical meristem was measured after 4 weeks.

### Callus induction from directly regenerated tissue

The secondary meristem, leaf, and petiole from the sterile, directly regenerated plants were used for callus induction. The leaf and petiole were cut into fragments of 10 mm and placed in callus induction media (CIM) containing 1 × MS base salt with 8.5 g/L agar, 30 g/L sucrose, and 0.1 mg/L NAA and supplied with different concentrations of 6-benzylaminapurine (6-BA, 1, 2 mg/L), kinetin (KT, 1, 2 mg/L), and thidiazuron (TDZ, 1, 2 mg/L), named as L1–L6 groups, respectively; L0 without cytokinin was used as control. About 18 leaf and petiole fragments were inoculated in each plastic cultivating pot (80 mm × 96 mm with breathable plastic cover) containing 50 mL of CIM. The cultivating bottle was placed in the same thermostatic chamber under two photoperiods of 16 h light/8 h dark and 24 h dark/d. Five pots were used for each treatment, and all of treatments was repeated three times. The induction rate of callus and the growth of explants were detected after 3 weeks.

### Induction and growth of adventitious buds

The callus from the secondary meristem, leaf, and petiole was inoculated in the screened meristem medium after 1 month of callus induction. Eight calluses (10 mm in diameter) from each material per container were placed in each culture pots for induction of adventitious buds. Five pots were used for each callus for induction of adventitious buds, and all of materials was repeated three times. The adventitious buds were divided into single bud to strengthen the shoots in the same medium after 30 days of induction. The inducting ratio of the adventitious buds was also determined.

### Root induction and plant acclimatization

After the plants were growth into 3 cm high, the rooting and plant acclimatization effect were evaluated through sterile solid media and improved hydroponics. The rooting media contained 1/2 × MS base salt with 8.5 g/L agar and 10 g/L sucrose supplied with different kinds and concentrations of auxin (M0–M3, Table 3). Plastic seeding boxes (365 mm × 230 mm × 110 mm, including lid and seeding plate and base plate) were used in hydroponics. A 1:1 mixture of perlite and vermiculite was used as the fixed material in the improved hydroponics. The level of culture liquid was maintained in 3 cm high, and the culture liquid contained 1/2 × MS base salt and supplied with different kinds and concentrations auxin (H1–H4, Table 3) but without agar and sucrose. H0 was supplied with distilled water and used as control. Number of regeneration buds per group was twelve, three replication of each group were laid. The seeding boxes and media were set in the same thermostatic chamber with vitro culture. The survival rate and effect of root induction were determined after 30 days.

### Evaluation of yield metric and main active ingredient

The virus-free plants were transplanted into soil in Zhongjiang, Sichuan, China after hardening–seeding in March, 2020 at the germination time of the cultivated plants. A land that has a flat terrain, smooth drainage, sunny terrain, no soil pollution, and never planted with *S. m*-SC was selected. The plants were alternately planted in ridges and watered daily with tap water for 1 month after transplantation.

The root and rhizome tissues were harvested separately from the virus-free plants and common plants after 10 months of culture for yield metric and main active evaluation. Root weight, main root number, main root length, and main root diameter per plant were measured.

The main active ingredient, salvianolic acid B, was assayed after drying by referring to Chinese pharmacopoeia (2020 edition). Main active ingredients were determined by HPLC (1260 Infinity II, Agilent Technologies, American).

### Data statistics and analysis

Data were analyzed using SPSS version 23.0 statistics and Duncan's test. One-way ANOVA was used for multiple comparisons.

## Supplementary Information

**Additional file 1.** **Figs.1** Virus detection results of diseased plants (D1~D17) of S.m.-SC by Das-ELISA test. **Figs.2** Virus detection results of regenerated plants of S.m.-SC by Das-ELISA test. **Figs.3** Casllus induction rate and growth status of SP and SL under two kinds light condition of 16 h light/ 8 h dark and 24 h dark/d.

## Data Availability

All data generated or analysis during this study is included in published article. The materials are available from the corresponding author on reasonable request.

## References

[1] Kum KY, Kirchhof R, Luick R (2021). Danshen (*Salvia miltiorrhiza*) on the global market: what are the implications for products' quality?. Front Pharm.

[2] Lai ZC, He JX, Zhou CX (2021). Tanshinones: an update in the medicinal chemistry in recent five years. Curr medicinal chemistry.

[3] Jung L, Kim H, Moon S (2020). Overview of *Salvia miltiorrhiza* as a potential therapeutic agent for various diseases: an update on efficacy and mechanisms of action. Antioxidants (Basel).

[4] Zhou J, Jiang YY, Chen H (2020). Tanshinone I attenuates the malignant biological properties of ovarian cancer by inducing apoptosis and autophagy via the inactivation of PI3K/AKT/mTOR pathway. Cell Prolif.

[5] Liang H, Kong Y, Chen W (2020). The quality of wild *Salvia miltiorrhiza* from Dao Di area in China and its correlation with soil parameters and climate factors. Phytochem Anal.

[6] Chen RS, Jiang M, Chen SM (2010). Drug production discrimination(Two). Trad Chinese Med and Clinic.

[7] Yu Y, Jiang YY, Wang L (2021). Comparative transcriptome analysis reveals key insights into male sterility in *Salvia miltiorrhiza* Bunge. PeerJ.

[8] Chen XH, Ye HZ, Yan JM. Investigation on the diseases of medicinal plants in Sichuan province and pathogen identification I. a list of diseases of main cultivated medicinal plants. Sout China Jour of Agri Sci. 2006.

[9] Ding YJ, Wu ZM, Xie XL (2003). Identification of pathogen from Danshen virus diseases in China. Chinese Trad and Herb Drugs.

[10] Alam MF, Banu MLA, Swaraz AM (2004). Production of virus free seeds using meristem culture in tomato plant under tropical conditions. J Plant Biot.

[11] Tània SP, Najet G, Rosa P (2017). Somatic embryogenesis from seeds in a broad range of Vitis vinifera L varieties: rescue of true-to-type virus-free plants. BMC Plant Biol.

[12] Yahyaoui E, Marinoni DT, Botta R, Ruffa P, Germanà MA (2021). Is it possible to produce certified hazelnut plant material in sicily? Identification and recovery of nebrodi genetic resources, in vitro establishment, and innovative sanitation technique from apple mosaic virus. Front Plant Sci.

[13] Tsai KL, Chen EG, Chen JT (2016). Thidiazuron-induced efficient propagation of *Salvia miltiorrhiza* through in vitro organogenesis and medicinal constituents of regenerated plants[J]. Acta Phys Plant.

[14] Koichiro M (1991). Tanshinone production in adventitious roots and regenerates of *Salvia miltiorrhiza*. J Nat Prod.

[15] Liang HW (2009). Tissue culture and plant regeneration of Salvia miltiorrhiza Bgefalba. J Anh Agri sci..

[16] Yan L, Zhang J, Chen H (2021). Genome-wide analysis of ATP-binding cassette transporter provides insight to genes related to bioactive metabolite transportation in *Salvia miltiorrhiza*. BMC Genomics.

[17] Ikeuchi M, Rymen B, Sugimoto K (2020). How do plants transduce wound signals to induce tissue repair and organ regeneration?. Curr Opin Plant Biol.

[18] Ikeuchi M, Ogawa Y, Iwase A (2016). Plant regeneration: cellular origins and molecular mechanisms. Development.

[19] Hai JW, Xiao YQ, Zhi CD (2020). WUSCHEL triggers innate antiviral immunity in plant stem cells. Science.

[20] Xue Z, Liu L, Zhang C (2020). Regulation of shoot apical meristem and axillary meristem development in Plants. Int J Mol Sci.

[21] Isah, T. De novo in vitro shoot morphogenesis from shoot tip-induced callus cultures of *Gymnema sylvestre* (Retz.) R.Br. ex Sm. Biological Research. 2019;52(1). 10.1186/s40659-019-0211-1. 10.1186/s40659-019-0211-1 PMC6339694 30660192

[22] Sugimoto C, Temman S, Kadokura R (2019). To regenerate or not to regenerate: factors that drive plant regeneration. Curr Opin Plant Biol.

[23] Skoog F, Miller CO (1957). Chemical regulation of growth and organ formation in plant tissues cultured in vitro. Symp Soc Exp Biol.

[24] Chong WD, Yan YY, Yu ML (2020). The regeneration of Acer rubrum L "October Glory" through embryonic callus. BMC Plant Biol.

[25] Zhang Y, Bozorov TA, Li DX (2020). An efficient in vitro regeneration system from different wild apple (*Malus sieversii*) explants. Plant Methods.

[26] Dobránszki E, Silva J (2011). Adventitious shoot regeneration from leaf thin cell layers in apple. Sci Hortic.

[27] Grant PP, Barril C, Schmidtke LM (2017). Light-induced changes in bottled white wine and underlying photochemical mechanisms. Crit Rev Food Sci Nutr.

[28] Liu F, Cui LJ, He G (2011). Dynamic changes in several effective components in different vegetative organs of Salvia miltiorrhiza Bge cultivars in different seasons. Plant Sci Jour..

[29] García F, Lluch R, Pineda C (2020). A highly efficient organogenesis protocol based on zeatin riboside for in vitro regeneration of eggplant. BMC Plant Biol.

[30] Shasmita K, Rai MK, Naik SK (2017). Exploring plant tissue culture in Withania somnifera (L) dunal: in vitro propagation and secondary metabolite production. Crit Rev Biot.

[31] Li QS, Yu P, Lai JR (2021). Micropropagation of the potential blueberry rootstock-*Vaccinium arboreum* through axillary shoot proliferation. Sci Hort.

[32] Benzle K, Cornish K (2017). Improved axenic hydroponic whole plant propagation for rapid production of roots as transformation target tissue. Plant Methods.

[33] Rahaman MM, Zwart RS, Thompson JP (2020). Constitutive and induced expression of total phenol and phenol oxidases in wheat genotypes ranging in resistance/susceptibility to the root-lesion nematode *Pratylenchus thornei*. Plants (Basel).

[34] Qu GW, Xie FX, Yue XD, et al. Distribution of salvianolic acid B and tanshinon II A in the root of *Salvia miltiorrhiza* Bge. Modern Chinese Medicine. 2005;7(1):11–13,17. 10.13313/j.issn.1673-4890.2005.01.005.

[35] Wei GF, Liu Q, Li J (2015). Distribution rule of active components in the roots of *Salvia miltiorrhiza* Bge. Shandong Science.

