# Supplementary material for: Development of a highly efficient virus-free regeneration system of Salvia miltiorrhiza from Sichuan using apical meristem as explants
Source: Plant Methods. 2022 Apr 18;18:50. doi: 10.1186/s13007-022-00872-4 (PMC9014595; doi:10.1186/s13007-022-00872-4)
Supplement: Supplementary file 1 — Additional file 1. Figs.1 Virus detection results of diseased plants (D1~D17) of S.m.-SC by Das-ELISA test. Figs.2 Virus detection results of regenerated plants of S.m.-SC by Das-ELISA test. Figs.3 Casllus induction rate and growth status of SP and SL under two kinds light condition of 16 h light/ 8 h dark and 24 h dark/d. [file 13007_2022_872_MOESM1_ESM.docx]

**Supporting information**

**Additional file 1：Figs.1** Virus detection results of diseased plants (D1~D17) of *S.m.*-SC by Das-ELISA test. **Figs.2** Virus detection results of regenerated plants of *S.m.*-SC by Das-ELISA test. **Figs.3** Callus induction rate and growth status of SP and SL under two kinds light condition of 16 h light/ 8 h dark and 24 h dark/d.

**
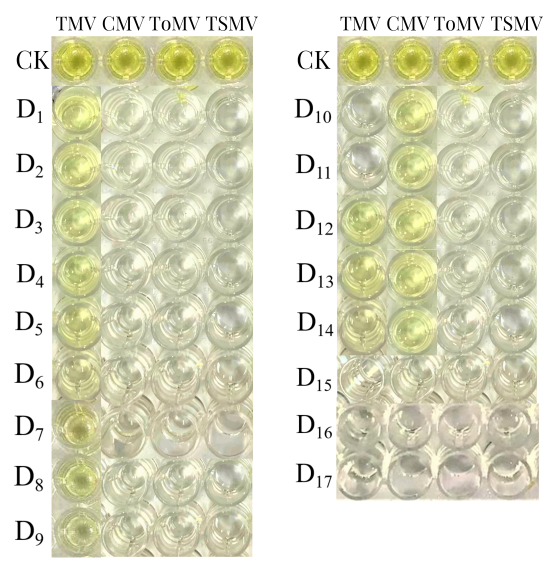
**

Figs.1 Virus detection results of diseased plants (D1~D17) of *S.m.*-SC by Das-ELISA test. CK: positive control, D1~D17: diseased plants. All plant samples were determined at least twice.


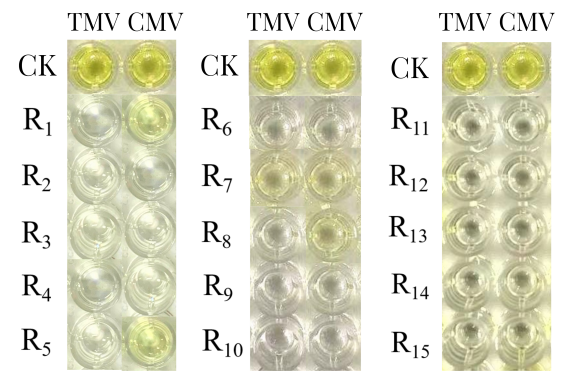


Figs.2 Virus detection results of regenerated plants of *S.m.*-SC by Das-ELISA test. CK: positive control, R1~R15: directly regenerated plants. R1, R5, R8: CMV positive; R7: CMV and TMV positive, other was negative. All plant samples were determined at least twice.


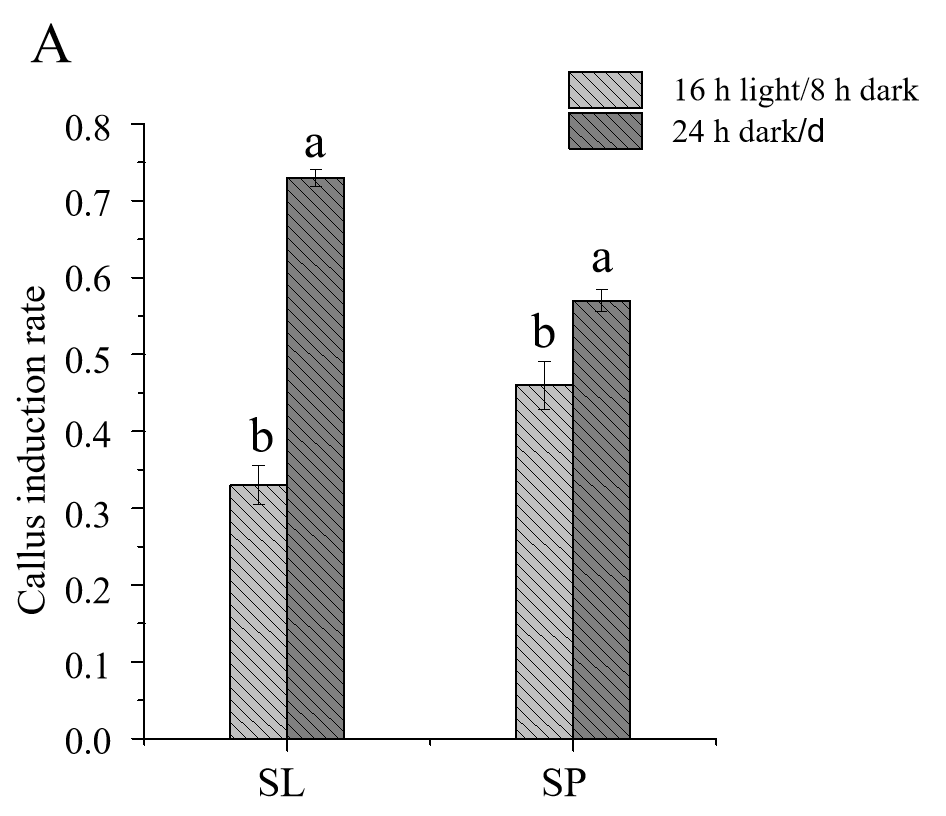

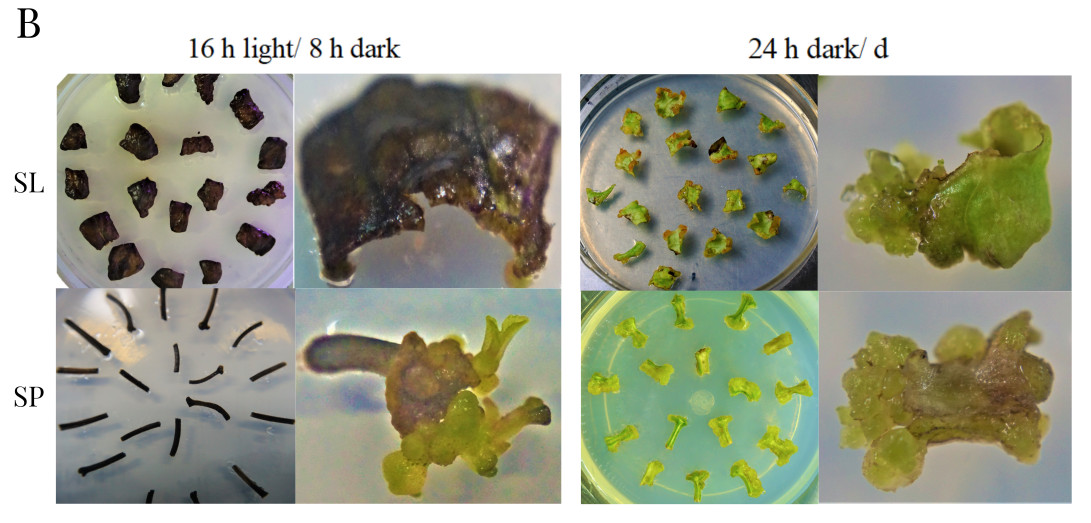


Figs.3 Callus induction rate and growth status of SP and SL under two kinds light condition of 16 h light/ 8 h dark and 24 h dark/d. **A:** callus induction rate of SL and SP under two kinds of light condition, namely, 16 h light/ 8 h dark and 24 h dark/d. **B:** Growth status of SP and SL on L2 media under two kinds of light condition, namely, 16 h light/ 8 h dark and 24 h dark/d. SL: secondary leaf, SP: secondary petiole. Different letters indicate significant differences among light condition for each group as determined by Duncan’s multiple range test followed by one-way ANOVA at the p<0.05 significant level. Number of secondary tissue inoculated per group was about eighteen; three replication of each group were laid. Data were collected after 3 weeks.
